# Targeting platelet-derived soluble CD40 ligand: a new treatment strategy for HIV-associated neuroinflammation?

**DOI:** 10.1186/1742-2094-10-144

**Published:** 2013-12-01

**Authors:** Donna C Davidson, Joseph W Jackson, Sanjay B Maggirwar

**Affiliations:** 1 Department of Microbiology and Immunology, University of Rochester School of Medicine and Dentistry, 601 Elmwood Avenue, Box 672, Rochester, NY 14642, USA

**Keywords:** Soluble CD40 ligand, HIV, HIV-associated neuroinflammation, HIV, Associated neurocognitive disorders, Platelets, Antiplatelet therapy

## Abstract

Human immunodeficiency virus type 1 (HIV) continues to be one of the most prevalent global health afflictions to date. The advent and introduction of combined antiretroviral therapy (cART) has made a significant impact on the course of infection. However, as patients are living longer, many HIV-associated illnesses are becoming prevalent among the infected population, especially those associated with chronic inflammation. Consistently, HIV-associated neuroinflammation is believed to be a major catalyst in the development of HIV-associated neurocognitive disorders (HAND), which are estimated to persist in approximately 50% of infected individuals regardless of cART. This dramatically underscores the need to develop effective adjunctive therapies capable of controlling this aspect of the disease, which are currently lacking.

We previously demonstrated that the inflammatory mediator soluble CD40 ligand (sCD40L) is elevated in both the plasma and cerebrospinal fluid of cognitively impaired infected individuals compared to their non-impaired infected counterparts. Our group, and others have recently demonstrated that there is an increasing role for this inflammatory mediator in the pathogenesis of HIV-associated neuroinflammation, thereby identifying this molecule as a potential therapeutic target for the management of HAND. Platelets are the major source of circulating sCD40L, and these small cells are increasingly implicated in a multitude of inflammatory disorders, including those common during HIV infection. Thus, antiplatelet therapies that minimize the release of platelet-derived inflammatory mediators such as sCD40L are an innovative, non-traditional approach for the treatment of HIV-associated neuroinflammation, with the potential to benefit other HIV-associated illnesses.

## Introduction

CD40 ligand (CD40L, formally known as CD154) is a type II membrane glycoprotein of the tumor necrosis factor (TNF) family that is found on activated T cells, B cells and platelets [1]. Classically, CD40L is a co-stimulatory molecule expressed on the surface of activated CD4 positive T cells. It binds to its receptor, CD40, on the surface of antigen-presenting cells to induce activation and enhance the expression of B7 molecules to promote T cell expansion. In B cells, CD40L binding induces proliferation and immunoglobulin class switching [1,2], while the absence of CD40L, as in X-linked hyper IgM syndrome, confers immunosuppression and lack of immunoglobulin class switching [3,4]. In addition to T cells, CD40L is also found on the surface of monocytes, macrophages, endothelial cells and platelets [2]. Cleavage of CD40L from the surface of cells produces a truncated, soluble form, sCD40L, which retains biologic activity and the ability to act as a cytokine [1,5,6]. Based on cellular distribution studies, platelets are believed to be almost the sole producers of sCD40L, responsible for approximately 95% of all sCD40L found in the plasma [7].

Beyond the co-stimulatory role of CD40L, this mediator also plays an important role in mediating inflammation. During an innate immune response, cytokines and chemokines are released by local cells at the site of insult. Subsequently, these molecules signal to monocytes to migrate to these areas, thus initiating an inflammatory response, which aids in wound healing and the clearance of dead or damaged cells. If there is vessel damage, the platelets encounter extracellular matrix proteins such as collagen, which induces the release of hemostatic mediators, including sCD40L, from these cells [7]. CD40 is constitutively expressed on endothelial cells. On ligation by sCD40L these cells become more conducive to monocytes that are recruited via a so-called inflammatory endothelial phenotype [8]. This phenotype has increased expression of adhesion molecules such as intracellular adhesion molecule 1 (ICAM-1) and vascular cell adhesion molecule 1 (VCAM-1), and is associated with the release of additional cytokines and chemoattractants [8,9]. Once recruited, monocytes can complex with platelets for further activation, or firmly adhere to the endothelium to extravasate through the vessel and into the tissue for the clearance of infection.

Inflammation is a delicate process that requires careful maintenance and balance; hence, dysregulation of this process can lead to various disease states. Following infection with human immunodeficiency virus type 1 (HIV), immune activation and inflammation are common, which are the result of both viral replication and cellular activation, and collectively lead to the release of a large number of pro-inflammatory mediators termed HIV effector molecules [10,11]. These host-derived and viral proteins are thought to be the main culprits responsible for overstimulating a wide range of cells, both in the central nervous system (CNS) and in the periphery, which perpetuates inflammation during infection. This, in turn, leads to the establishment of a chronic inflammatory disease state [10]. HIV effector molecules persist despite combined antiretroviral therapy (cART), and contribute to the development of many HIV-associated illnesses, including HIV-associated neuroinflammation.

CD40L has been implicated previously in HIV-associated immune activation and inflammation, as it has been demonstrated that CD40-CD40L signaling suppresses production of the antiviral response protein interferon α in plasmacytoid dendritic cells, leading to an increased production of cytokines in peripheral blood mononuclear cells [12]. Furthermore, this lack of an interferon α-mediated antiviral response may contribute to increased opportunistic infections and the immune reconstitution inflammatory syndrome (IRIS) in some HIV patients [13]. This further supports the notion that the establishment of a chronic inflammatory state in HIV-infected individuals is due to an increase in HIV effector molecules such as sCD40L, and highlights the potential for identifying candidate HIV effector molecules as novel therapeutic targets.

CD40L has been implicated in numerous inflammatory conditions, such as cardiovascular disease [2,7], atherosclerosis [14], inflammatory bowel syndrome [15], fibrosis [16] and many more, in addition to HIV infection [12,13,17]. For example, patients with type 1 diabetes have been found to have elevated levels of circulating sCD40L, which was implicated in stimulating monocytes to express a more pro-inflammatory phenotype [18], further demonstrating a role for this molecule in the persistence of a chronic inflammatory state. Interestingly, CD40L signaling is also implicated in several neurodegenerative disorders including cerebral malaria [19] and Alzheimer’s disease [20,21]. Accordingly, in a mouse model of Alzheimer’s disease, Tan *et al.* observed that mice deficient in CD40L had reduced astrocytosis and microgliosis compared to mice expressing CD40L [21]. Collectively, these studies implicate CD40-CD40L signaling as a potential therapeutic target not only for persistent inflammatory diseases, but also for neuroinflammatory disorders.

In many of these disorders it is believed that the excessive interaction of platelet-derived sCD40L with CD40 on the surface of endothelial cells induces an inflammatory endothelial cell phenotype, as discussed above, and ultimately aberrant inflammation, tissue infiltration and cellular damage. Interestingly, increased infiltration of the CNS by activated leukocytes is widely believed to be the largest contributing factor in the development of HIV-associated neuroinflammation, due to the excessive release of HIV effector molecules within the CNS by these activated cells, and the development of a progressively neurotoxic environment [22]. Activation and dysregulation of brain microvascular endothelial cells (BMVECs), which form the blood–brain barrier (BBB), have been reported in the context of HIV and ultimately result in the deterioration of the barrier, and in turn, facilitate the recruitment and transmigration of activated or infected leukocytes through the BBB, exceeding that which is considered routine [23].

It has recently been purported that sCD40L may have a larger role than previously thought in the pathogenesis of HIV-associated neuroinflammation and the subsequent development of HIV-associated neurocognitive disorders (HAND). Our group has observed that plasma and cerebrospinal fluid concentrations of sCD40L are elevated in HIV-infected, cognitively impaired individuals [17] compared to infected, non-cognitively impaired patients. Furthermore, CD40-CD40L signaling has been implicated in HIV-associated neuroinflammation previously: Ramirez *et al.* observed an increase in CD40 expression on BMVECs from patients who had succumbed to infection and been diagnosed with HIV encephalitis compared to BMVECs from control brains [24], indicating that endothelial cells in patients with HAND-related complications are highly responsive to excess amounts of sCD40L. Consistently, it has been reported that microglia cells derived from HIV-encephalitic patients have increased expression of CD40, further demonstrating the involvement of this signaling pathway in these disorders [25].

Additional studies by our group, using both wild-type (WT) and CD40L-deficient mice, have recently demonstrated that excess sCD40L is induced by the HIV Tat protein in a manner that promotes increased BBB permeability and enhanced attachment of monocytes to the brain microvasculature *in vivo*[26]. This effect was found to be mediated by platelet activation, and the subsequent release of sCD40L, since depletion of platelets in WT mice prior to Tat treatment led to an amelioration of the Tat-induced BBB permeability, in the same manner observed in CD40L-deficient animals [26]. While there are many factors, both host derived and viral, that orchestrate the breakdown of the BBB during HIV infection [25,27-30], we observed a drastic effect mediated by platelet-derived CD40L. Collectively, these results suggest that the CD40L/CD40 pair has a large role in the progression of HAND, and provide evidence that excessive platelet activation may be contributing to a multitude of other inflammatory disorders.

Consistent with this notion, there is an expanding view of the role for platelets in the pathogenesis of HIV, as they interact with a number of immune cells and contribute to the breadth of pro-inflammatory mediators found during infection [31]. Aberrant platelet activation is increasingly recognized as a major contributor to a number of inflammatory conditions, including atherosclerosis, diabetes and arthritis. In the context of HIV, it has been found that platelets circulate in a more activated state within infected individuals [32], and HIV infection is associated with the release of molecules known to activate platelets, such as platelet-activating factor [33]. Ironically, thrombocytopenia is frequently diagnosed in HIV-infected individuals, suggesting that the contribution of platelets to HIV-associated illnesses would be low, given the low numbers of these cells detected in these patients. However, platelet activation in the periphery is followed by rapid removal of these cells from circulation, thereby indicating that a low platelet count follows overstimulation of platelets during HIV infection, followed by their clearance. Furthermore, activated platelets may be sequestered in cellular aggregates during HIV infection [34,35], which could also contribute to decreased detection of these cells in complete blood cell counts of infected individuals. In line with this, it has been demonstrated that platelet decline during HIV infection is associated with the onset of neurological impairment [36,37], thus indicating that platelet activation and their subsequent removal from circulation precedes the onset of neurological symptoms and has a large role in the pathogenesis of HAND.

Although the introduction of combination antiretroviral therapy (cART) has made a tremendous impact on the course of HIV as well as HAND, there remains a critical lack of effective adjunctive therapies for the management of the neuropathological aspect of these diseases. With over 50% of cART-receiving patients still suffering from some form of cognitive impairment [38], it is clear that this is an area that cannot be ignored. In general, it is widely believed that one of the largest contributing factors in the initiation of HAND is the breakdown of the BBB, which allows infiltration of the CNS by activated or infected macrophages that perpetuate this inflammatory disease. Hence, identifying the underlying mechanisms that contribute to this breakdown, and thus drive the pathogenesis of HAND, would reveal novel targets for therapeutic intervention. Antiplatelet therapies that minimize the release of platelet-derived inflammatory mediators such as sCD40L may therefore prove to be a worthy avenue of pursuit in identifying novel treatment strategies for HAND. In addition, given the involvement of sCD40L in the dysregulation of multiple immune response mechanisms in the context of HIV [39,40], this approach may have the added benefit of modulating both HAND and other HIV-associated inflammatory disorders.

### Platelets and vascular permeability

Platelets are small, approximately 3 μm, anucleate cells involved in hemostasis, the process of blood clotting, and inflammation. Although they do not have a nucleus, platelets do contain most of the classical cellular components, and have the ability to process precursor mRNA and translate mRNA into proteins [41,42]. Platelets are the first cells to respond to vascular injury, interacting with endothelial cells to form a plug over the wound, and they aid in leukocyte recruitment via the release of inflammatory mediators [8]. Normal platelet–endothelial cell interactions at the site of an injury are similar to those seen in leukocyte rolling; platelets roll along endothelial cells and form loose contacts upon interaction with selectins on the surface of activated endothelial cells or with extracellular matrix components such as collagen or fibrinogen [8,9]. These interactions activate the platelet and induce firm adhesions through integrins on both the platelet and the endothelial cell surfaces, which in turn causes platelet aggregation, cytoskeletal rearrangement and a signaling cascade that ultimately leads to the release of cytokines and chemoattractants from both platelets and endothelial cells [8,43]. This process is necessary for normal wound healing and hemostasis; however, as outlined above, dysregulation or overstimulation of this process can lead to many inflammatory complications. Similarly, low platelet counts, or thrombocytopenia, is also cause for alarm, as it can induce hemorrhage. Thus, a delicate balance of stimulation and attenuation of signaling within these cells is vital for proper homeostasis.

In the event that this balance is disrupted, via the induction of an excessive inflammatory BMVEC phenotype by platelet-derived sCD40L within the plasma of HIV-infected individuals [17,44], it is plausible that sCD40L largely contributes to the increased permeability of the BBB seen during HAND, thereby increasing monocyte traffic at the BBB and invasion of the CNS by pro-inflammatory cells [26]. However, this implies a contradictory role for platelets in vessel integrity during inflammatory disease. This is an interesting thought given that the classical role of platelets is to prevent vessel leakage by forming a plug over areas of insult in an effort to minimize vessel and tissue damage [45]. It is clear that a basal level of endothelial cell permeability is necessary for the proper exchange of nutrients, ions and water, and it allows leukocytes access to injured tissues; thus, platelets aid in the development of an environment at the endothelium that is conducive to a modest amount of vascular permeability. Therefore, upon dysregulation of this system, it is likely that aberrant activation of platelets could intensify this process in a pathological manner, thereby corroborating the observed effect that the absence of platelets can ameliorate vascular permeability in certain circumstances.

**Figure 1:**
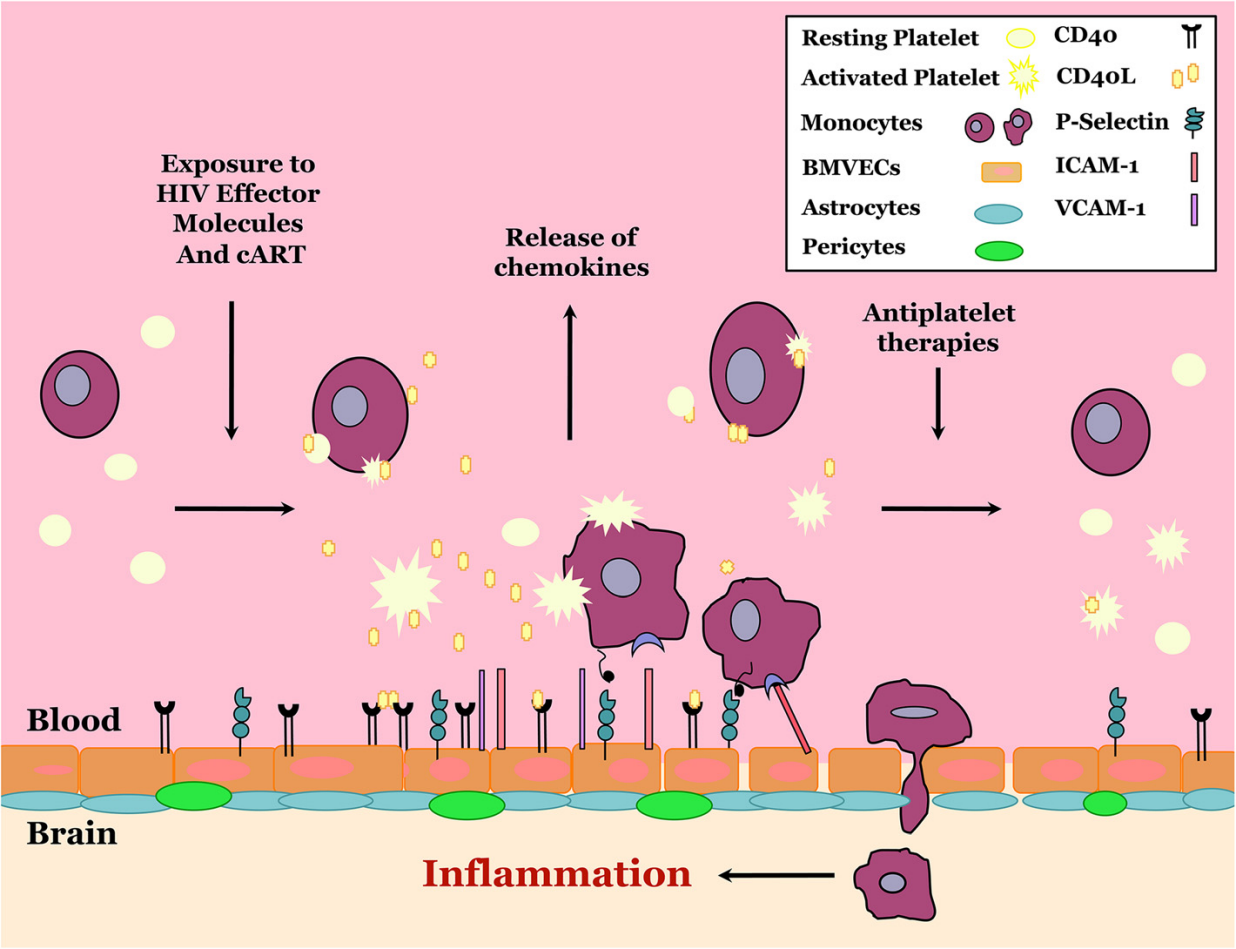
**Working model and overall summary.** Upon exposure to HIV effector molecules, both viral and host derived, platelets become aberrantly activated and release an excess of soluble CD40 ligand (sCD40L). This sCD40L can then bind to its receptor, CD40, on the surface of brain microvascular endothelial cells (BMVECs), inducing an inflammatory endothelial cell phenotype. Features of this phenotype include upregulation of cellular adhesion molecules such as P-selectin, ICAM-1 and VCAM-1, as well as the release of the chemoattractant MCP-1. Subsequently, leukocytes are recruited to the endothelium, where they are able to adhere and migrate through the barrier and into the central nervous system, thus stimulating a pro-inflammatory, excitotoxic environment. Therefore, inhibition of sCD40L release from platelets via antiplatelet therapies has the potential to attenuate this process. BMVEC, brain microvascular endothelial cell; cART, combined antiretroviral therapy; ICAM-1, intracellular adhesion molecule 1; sCD40L, soluble CD40 ligand; VCAM-1, vascular cell adhesion molecule 1.

As previously mentioned, platelets circulate in a more activated state within HIV-infected individuals [32,46], and HIV infection is associated with an increase in various markers of platelet activation, such as soluble P-selectin, sCD40L and platelet-monocyte complexes [17,35,44,46]. Furthermore, HIV is associated with an increased risk of thrombotic complications and vascular disease, of both the cardiovascular and cerebrovascular systems [47-50]. Thus, the inflammatory environment observed during HIV infection seems to support the dysregulation of platelets: it causes excessive activation and the subsequent release of sCD40L, and contributes to vascular permeability, contrary to the classical role of platelets (Figure 1).

Consistent with this notion, there is an expanding breadth of knowledge to suggest that platelets play a role in numerous inflammatory disorders, including those that are associated with vascular permeability. Interestingly, Cloutier *et al.* recently demonstrated that synovial vascular permeability observed in a murine experimental arthritis model was abrogated when platelets were depleted prior to the onset of arthritis [51]. The authors note that this was an unexpected finding due to the classical role of platelets in vessel maintenance, and may be the result of the severe inflammatory environment in the experimental arthritis model and the subsequent overstimulation of platelets [51]. Similarly, several reports note that antibody-mediated depletion of platelets can attenuate vascular permeability and leukocyte infiltration in an acute lung injury mouse model, via decreased endothelial cell adhesion molecule expression and mediator release [52], and in a cecal ligation and puncture (CLP) murine model of sepsis, due to a decrease in both chemokine release and platelet-leukocyte interactions [53]. Consistently, using both platelet depletion and CD40L-deficient animals, Rahman *et al.* identified platelet-derived CD40L as a driving force in the pathologic recruitment and infiltration of leukocytes into the lung in the CLP mouse model, thereby aiding in the development of lung injury and edema [54]. Furthermore, Lapchak *et al.* also employed both platelet depletion and CD40- or CD40L-deficient mouse models of mesenteric ischemia/reperfusion injury and observed lung damage only when platelets, CD40, or CD40L were present, whereas platelet-depleted mice and those deficient in CD40 or CD40L had a marked absence of vessel damage [55].

In line with this, platelet activation and the CD40/CD40L axis have also been reported to have induced vascular permeability in ischemia/reperfusion injury in the brain [56], as well as other disorders of the CNS, notably cerebral malaria [19,57,58]. These studies have taken advantage of both platelet depletion and/or CD40- or CD40L-deficient mouse models to demonstrate that CD40L signaling is required for the breakdown of the BBB and consequential tissue damage. These results are consistent with our previous findings, and further highlight the unconventional role of platelet activation and the subsequent release of sCD40L in the progression of diseases known to be exacerbated by vascular permeability in both the periphery and the CNS.

### Antiplatelet therapies for the management of HIV-associated neuroinflammation

The identification of novel roles for sCD40L in inflammation and illness, such as those described above, has highlighted the need to develop novel clinical approaches to inhibit this platelet-derived mediator. Traditionally, the therapeutic targeting of sCD40L has presented quite a challenge due to the co-stimulatory role of this mediator in both innate and adaptive immunity, as it is required for B cell class switching during humoral immune responses. As such, classical treatments such as cyclosporine A or anti-CD40L monoclonal antibodies that cannot distinguish between surface-expressed co-stimulatory CD40L on T cells and soluble platelet-derived CD40L, can confer immunosuppression, and therefore are not suitable for use in HAND. However, the use of antiplatelet agents that can attenuate the release of sCD40L from platelets, while leaving surface-expressed co-stimulatory CD40L unaltered, would prevent the negative effects on the humoral immune response, and thus represents an attractive alternative therapeutic strategy with broad implications for a wide range of inflammatory disorders. To this end, it is an interesting notion that antiplatelet agents, which are traditionally used for the management of cardiovascular disease or inflammation in the periphery, could be used to treat neurological disorders. However, this strategy has benefits that are pleiotropic, in that it would also serve to reduce the risk of cardiovascular, thrombotic and other inflammatory-associated complications that are common in HIV-infected individuals.

There are currently numerous strategies for targeting platelet activity, most of which are centered on the use of aspirin, ADP receptor antagonists and GPIIb-IIIa (αIIbβ3) antagonists, and they have largely been studied in the context of cardiovascular disease.

#### Aspirin

Aspirin has been studied as an antiplatelet agent for decades, and there is an incredible wealth of knowledge on the clinical utility, as well as the drawbacks, of this drug. The effects of aspirin are mediated by its attenuation of prostaglandin synthesis from arachidonic acid, via the irreversible inhibition of cyclooxygenase 1 and 2 (COX-1 and COX-2, respectively) [59]. Interestingly, while aspirin is recommended for the management or prevention of numerous thrombotic-associated diseases, such as acute myocardial infarction, acute ischemia/reperfusion injury and unstable angina [60], aspirin has been shown to have little or no effect on the surface expression and subsequent release of CD40L from platelets [61,62]. Thus, when considered with the results reviewed herein, it seems aspirin may not be the most ideal candidate for use as an adjunctive therapy during HIV infection. However, it was recently demonstrated in a small pilot study that low-dose daily aspirin for 1 week can attenuate platelet and immune activation in HIV-infected individuals [63], further highlighting the potential utility of aspirin or other antiplatelet therapies to modulate HIV-induced inflammation.

#### GPIIb-IIIa antagonists

In contrast to aspirin, GPIIb-IIIa antagonists have been shown to control levels of sCD40L, which is not surprising given that GPIIb-IIIa is known to regulate the release of sCD40L from the surface of platelets [64]. Currently, three GPIIb-IIIa antagonists (abciximab, tirofiban and eptifibatide) are approved for clinical use for the treatment of thrombotic complications, and several reports indicate that these drugs are efficient at inhibiting the release of sCD40L from platelets in patients with various forms of cardiovascular disease [61,65,66]. During platelet aggregation, fibrinogen binds to GPIIb-IIIa, which can act as a bridging molecule in the formation of platelet aggregates, and this can lead to further platelet activation, degranulation and mediator release. Indeed, eptifibatide has also been found to regulate the expression of P-selectin on the surface of platelets [61]. Therefore, inhibition of GPIIb-IIIa not only attenuates the release of sCD40L from platelets, but also other pro-inflammatory mediators that are implicated in inflammatory diseases. Heeschen *et al.* demonstrated that treating patients suffering from acute coronary syndromes and have elevated levels of sCD40L with abciximab reduced the risk of death or myocardial infarction, clearly illustrating the impact that modulating mediator release from activated platelets can have on inflammatory diseases [66]. Although relatively little is currently known about the effects of these drugs on T and B cell responses, especially in the context of HIV infection, GPIIb-IIIa is a platelet-specific integrin, suggesting that off-target effects would be minimal.

#### ADP receptor antagonists

ADP receptor antagonists, such as clopidogrel, have been implicated in CD40L control *in vivo*[67], as ADP is known to induce CD40L surface expression and release from platelets; however, there is limited evidence on whether these drugs are able to control CD40L levels in the context of inflammatory disease. At least one study found that clopidogrel was able to attenuate plasma levels of sCD40L in immunosuppressed renal transplant patients, who are prone to platelet-associated inflammatory complications [68], implying that further studies with clopidogrel may be warranted for the management of inflammation in the context of HIV.

#### Phosphodiesterase inhibitors

An alternative antiplatelet agent that has been shown to reduce the activation-induced release of sCD04L from platelets is dipyridamole [69,70]. Although several mechanisms of action have been identified for this drug, the major way in which it inhibits platelets is via phosphodiesterase (PDE) inhibition. This ultimately causes the accumulation of the cyclic nucleotides cAMP and cGMP within platelets, which exerts an inhibitory effect [71]. Interestingly, PDE inhibitors may have multiple benefits for HAND in addition to controlling the release of sCD40L, as it was recently demonstrated that inhibition of PDE type 5, using the PDE inhibitor gisadenafil, could restore alterations in HIV Tat-induced cerebrovascular pathology and changes in cerebral blood flow in a mouse model of HIV neuroinflammation [72]. Furthermore, ibudilast, an alternative PDE inhibitor, attenuated Tat-induced inflammatory responses in a murine microglial cell line [73] and it has been shown to control the release of sCD40L from platelets [74]. Taken together, these results suggest that dipyridamole and other PDE inhibitors may be extremely beneficial in controlling HIV-induced inflammation and platelet activation, as well as neuroinflammation.

#### Statins

In addition to classical antiplatelet agents, other classes of drugs, notably statins and glitazones, can modulate CD40L signaling, and like antiplatelet agents they are utilized for the management of cardiovascular diseases. Statins, such as atorvastatin and simvastatin, inhibit 3-hydroxy-3-methylglutaryl–coenzyme A (HMG-CoA) reductase (HMGR), which is involved in a crucial step in the biosynthetic pathway that ultimately gives rise to cholesterol, and thus, they are used clinically to lower levels of low-density lipoprotein cholesterol [75]. In addition, these drugs can reduce the expression of various types of pro-inflammatory mediators such as cytokines, chemokines and adhesion molecules [76]. In the context of CD40L signaling, several studies have revealed that treating endothelial cells with various statins significantly reduces cytokine-induced expression of CD40 [77-79]. Consistently, Schonbeck *et al.* demonstrated in a small pilot study that a 6-month treatment with statins significantly reduced plasma levels of sCD40L in patients with atherosclerosis, to similar levels as those of healthy controls [77]. Furthermore, in a larger study, 110 patients with familial hypercholesterolemia who received statin therapy for 2 years had significantly lower plasma sCD40L concentrations than at baseline [80]. The mechanism by which statins decrease sCD40L *in vivo* remains to be determined; however, when considered with the results presented here, it is likely that statins are capable of inhibiting platelets, either directly or indirectly. In addition, the observed ability of statins to decrease CD40 on endothelial cells would be advantageous for the treatment of disorders associated with vascular permeability, such as HAND.

Unfortunately, despite a relatively high number of HIV-infected patients receiving statins for the control of hyperlipidemia due to cART [81,82], there is a lack of reported data on the incidence of HAND and neurological impairment in the statin-treated HIV-infected population, as well as the effect of statins on HAND. The potential utility of these compounds requires further investigation in the context of HIV.

Interestingly, statins have been shown to modulate T cell activation by inhibiting the activation-induced expression of adhesion molecules [76,83], and they can downregulate chemokine receptor expression on both B cells and T cells, thereby dampening inflammatory responses [83]. This immune-dampening effect of statins has also been observed in the context of HIV infection [84]. Furthermore, several reports have demonstrated that statins can decrease HIV replication by blocking cellular components that promote infectivity, such as host membrane proteins acquired by the virus that aid in attachment and the propagation of infection [85-87]. Although some statins can interact with several antiretrovirals, others are indicated for HIV-infected individuals for the management of cardiovascular disease [88] and dyslipidemia [81,82]. Therefore, with proper clinical assessment and individual consideration, statins may be safe and well tolerated during treatment for HIV infection. Collectively, these studies indicate that statins may be effective at mediating CD40-CD40L signaling, thus controlling HIV-associated inflammation.

#### Glitazones

Glitazones are a relatively new class of drugs that are used to treat type 2 diabetes mellitus and include the drugs troglitazone and rosiglitazone. A number of small studies involving patients with diabetes have shown that several of these drugs can significantly reduce sCD40L levels [89,90]; however, these drugs have been associated with hepatotoxicity and an increased risk of cardiovascular disease, and thus, may not be suitable for use as an adjunctive therapy in HIV infection.

#### Glycogen synthase kinase 3β inhibitors

The drugs discussed thus far are fairly classical examples of antiplatelet agents. While these drugs have obvious potential implications for the modulation of CD40L signaling beyond their current development as therapeutics for use in cardiovascular disease, there may be additional alternative methods for inhibiting platelet activation. Our group recently revealed the novel antiplatelet activity of valproic acid (VPA), which limits the release of sCD40L from platelets. It has promising clinical benefits for HIV-infected individuals [91] via the decreased plasma levels of sCD40L. This effect was due to the ability of VPA to attenuate platelet cytoskeletal rearrangement via inhibition of glycogen synthase kinase 3β (GSK3β) [92], as rearrangement is a necessary step in the translocation of CD40L to the surface of platelets for subsequent cleavage. Similarly, treating platelets with lithium, another GSK3β inhibitor, also halted cytoskeletal rearrangement. The idea of utilizing GSK3β inhibitors in the treatment of HAND is not new [91,93-95]; however, demonstrating that these inhibitors are capable of modulating platelet mediator release is a novel finding, and this is a desirable characteristic for a candidate adjunctive therapy for HAND. Indeed, patients receiving VPA for the treatment of mood disorders and epilepsy are not immunosuppressed, and hence VPA can modulate the effects of sCD40L without interfering with surface-expressed co-stimulatory CD40L. It would be advantageous if further studies could delineate the potential reduction in thrombotic complications within the periphery in the presence of GSK3β inhibitors.

Our group was the first to report that VPA could abrogate HIV-associated neurotoxicity both *in vitro* and *in vivo*[96,97]. In a controlled pilot patient study, we demonstrated a trend toward improved cognitive performance, as well as improvements in measures of brain metabolism [91]. These results highlight the potential benefits of this drug as an adjunctive therapy for HAND. Interestingly, some reports have suggested that VPA may not be well suited for use during HIV infection, as it is a histone deacetylase inhibitor and therefore may be capable of activating latent viruses, and thus, increasing viral replication. However, a small retrospective case–control study did not find that VPA was associated with an increase in viral load or HIV disease progression [98]. Moreover, several studies have utilized this characteristic of VPA in patients receiving this drug in combination with high doses of cART in an effort to deplete latent viral reservoirs, with some success [99,100]. Although there is evidence that VPA may affect the metabolism of some concomitant medications, it is noteworthy that co-administration of VPA with efavirenz or lopinavir does not alter the plasma concentrations of these drugs [101], thus substantiating the potential efficacy of VPA as an adjunctive therapy for HIV infection.

Several other reports have noted adverse side effects associated with long-term use of VPA [102,103]; however, many of these are case specific and vary depending on risk factors. The widespread clinical use of this drug has demonstrated its safety and tolerability, which make it an attractive therapeutic candidate for the management of HAND. Furthermore, the common clinical use of both VPA and lithium for the treatment of mood disorders and epilepsy, and the relatively low cost of these drugs, suggests that their implementation for an alternative use would be met with relative ease. Given the history of these drugs in the context of HAND [91,93,96,101], it seems that they, and alternative small molecule inhibitors of GSK3β, should not be ignored as candidates for adjunctive therapies during HIV infection.

Cognitive impairment persists in an estimated 50% of HIV-infected individuals regardless of cART [38], which underscores the inability of these therapeutic regimens to control this aspect of the disease. Moreover, it is now apparent that these drugs themselves may contribute to the development of these disorders via induction of sCD40L [104]. We found that non-nucleoside reverse transcriptase inhibitors (NNRTIs) can directly activate platelets, while other classes of antiretrovirals do not have this ability. This is an undesirable effect of cART given that these drugs are already relatively toxic. They can induce the activation of other cell types in addition to platelets [105,106], thus exacerbating the already chronically inflamed state. Interestingly, VPA was able to attenuate the release of sCD40L in both NNRTI-treated isolated human platelets via GSK3β inhibition, and in HIV-infected individuals receiving cART including a NNRTI [104]. The advantages of cART in the management of HIV infection are undeniable, and thus, these results further emphasize the value of adjunctive therapies such as VPA, which can offset the confounding inflammatory effects of cART.

## Concluding remarks

In light of the recent observation that more than half of HIV-infected individuals will develop some form of neurocognitive impairment regardless of antiretroviral therapy [38], it seems apparent now more than ever that novel therapeutic interventions are lacking and in dire need. Although the implementation of cART has shifted the severity of HAND to more mild forms, it has obviously failed to eradicate these disorders, thus implying that adjunctive therapies are a necessary therapeutic strategy. Hence, identifying novel targets, such as platelet-derived sCD40L, is an essential step towards improved patient care. As with all drugs, there are pros and cons associated with each of those described here; however, there is clear evidence to suggest that potential candidates for the management of HAND remain unexplored and may prove fruitful in the development of adjunctive therapies not only for HAND, but also for other pro-inflammatory HIV-associated illnesses.

## Abbreviations

BBB: Blood–brain barrier; BMVEC: Brain microvascular endothelial cell; cART: Combined antiretroviral therapy; CLP: Cecal ligation and puncture; CNS: Central nervous system; COX: Cyclooxygenase; GSK3β: Glycogen synthase kinase 3β; HAND: HIV-associated neurocognitive disorder; HIV: Human immunodeficiency virus type 1; ICAM-1: Intracellular adhesion molecule 1; NNRTI: Non-nucleoside reverse transcriptase inhibitors; PDE: Phosphodiesterase; sCD40L: Soluble CD40 ligand; VCAM-1: Vascular cell adhesion molecule 1; VPA: Valproic acid; WT: Wild-type.

## Competing interests

The authors declare that they have no competing interests.

## Authors’ contributions

DCD performed an extensive literature review, interpreted data, wrote the manuscript and created the figure. JWJ and SBM made substantial contributions to the conception and design of the manuscript, and they contributed to manuscript revisions. All authors read and approved the final manuscript.
